# Mannan oligosaccharide requires functional ETC and TLR for biological radiation protection to normal cells

**DOI:** 10.1186/s12860-018-0161-4

**Published:** 2018-06-27

**Authors:** Sweta Sanguri, Damodar Gupta

**Affiliations:** Division of Capacity Enhancement and Product Induction, Institute of Nuclear Medicine & Allied Sciences, Defence Research and Development Organization, Ministry of Defence, Brig. S.K. Mazumdar Marg, Timarpur, Delhi, 110054 India

**Keywords:** TLRs, Mannan oligosaccharide, Mitochondria, Radiation protection, ROS, Mitochondrial membrane potential, NFκB

## Abstract

**Background:**

Low LET Ionizing radiation is known to alter intracellular redox balance by inducing free radical generation, which may cause oxidative modification of various cellular biomolecules. The extent of biomolecule-modifications/ damages and changes in vital processes (*viz.* cellular homeostasis, inter−/intra-cellular signaling, mitochondrial physiology/dynamics antioxidant defence systems) are crucial which in turn determine fate of cells.

**Results:**

In the present study, we expended TLR expressing (normal/ transformed) and TLR null cells; and we have shown that mannan pretreatment in TLR expressing normal cells offers survival advantage against lethal doses of ionizing radiation. On the contrary, mannan pretreatment does not offer any protection against radiation to TLR null cells, NKE ρ° cells and transformed cells. In normal cells, abrupt decrease in mitochondrial membrane potential and endogenous ROS levels occurs following treatment with mannan. We intend to irradiate mannan-pretreated cells at a specific stage of perturbed mitochondrial functioning and ROS levels to comprehend if mannan pretreatment offers any survival advantage against radiation exposure to cells. Interestingly, pre-irradiation treatment of cells with mannan activates NFκB, p38 and JNK, alters mitochondrial physiology, increases expression of Cu/ZnSOD and MnSOD, minimizes oxidation of mitochondrial phospholipids and offers survival advantage in comparison to irradiated group, in TLR expressing normal cells.

**Conclusion:**

The study demonstrates that TLR and mitochondrial ETC functions are inevitable in radio-protective efficacy exhibited by mannan.

## Background

Progression in nuclear science results in proportionate exponentiation of the approaches with which it can be exploited for well being of the world. Contrarily, it also infringes threat of relatively greater order when mishandled (terrorist attacks) or in case of mishap (accidents such as those that occurred in Chernobyl and Fukushima). The development of countermeasure(s) to combat the potentially catastrophic events of radiation exposure is indispensable, which otherwise can results in mass causality, severe health consequences and/or lethality of organisms. Several radiation countermeasure agents’ *viz.* antioxidants, cytokines, toll like receptor (TLR) agonists, free radical mimetic agents etc. have been screened and explored intensively for their radio-protective efficacy [1–7]. In spite of the profuse endeavor, the world still have few molecules like (G-CSF, GM-CSF) that can be used as radiation countermeasure agent, that too for alleviating marrow acute radiation syndrome (ARS) only [6–10]. The others in-use have potential side effects associated with them and therefore numerous approaches are currently being enforced to discover a countermeasure agent, which is effective, and concomitantly safe [6]. TLRs are integral components of innate immune signaling, which helps in recognition of pathogens, molecular patterns and/or danger signals [11]. TLRs consist of highly conserved motifs and are specific for their ligands [9, 12]. Activation of these TLR related pathways might be exploited to study the ability of various TLR ligands in modification of radiation response. Recently, several agonists of TLRs have been shown to possess protective efficacy against lethal effects of ionizing radiation and are currently under different stages of development as radiation countermeasure agent for ARS [4, 6, 7, 9]. Most of these have been screened for their ability to activate NFκB pathway and reduce radiation-induced cell death in various tissues [4]. In the present investigation we tried to utilize properties of mannan oligosaccharide (MOS), a known TLR agonist both on normal and transformed cells to understand changes in biological radiation responses and radiation protection. MOS is long known for its gastrointestinal and immunological responses in several living organisms including, farm animals, pigs, dogs, cattle’s, fishes, chicken etc. [13–16]. There are several reports of improved health, growth status, enhanced performance, resurgence of the systemic and local immune system in animals [15, 17–19]. It has also been shown to stimulate epithelial barrier structure and functionality of intestinal mucosa [20]. Mannan has also been reported to possess anti-oxidative, anti-genotoxic and anti-mutagenic properties [21, 22]. Furthermore, mannan is known to possess anti-proliferative effects against several tumor cell lines and solid tumors [23, 24]. Recently, a novel pathway linking innate immune signaling to mitochondria has been elucidated, showing evidence of direct communication between TLRs and mitochondria [12]. Moreover, mannan pretreatment to normal cells were found to restore the radiation induced changes in mitochondrial dynamics in normal cells [25]. In the present study we have shown that, mannan mediated alterations in mitochondrial physiology in immortalized normal cells reduces biological effects of γ-radiation and enhances the cell survival.

## Results

### Mannan mediated activation of NFκB and modification of MMP (ΔΨm) in association with ROS generation

**Fig. 1:**
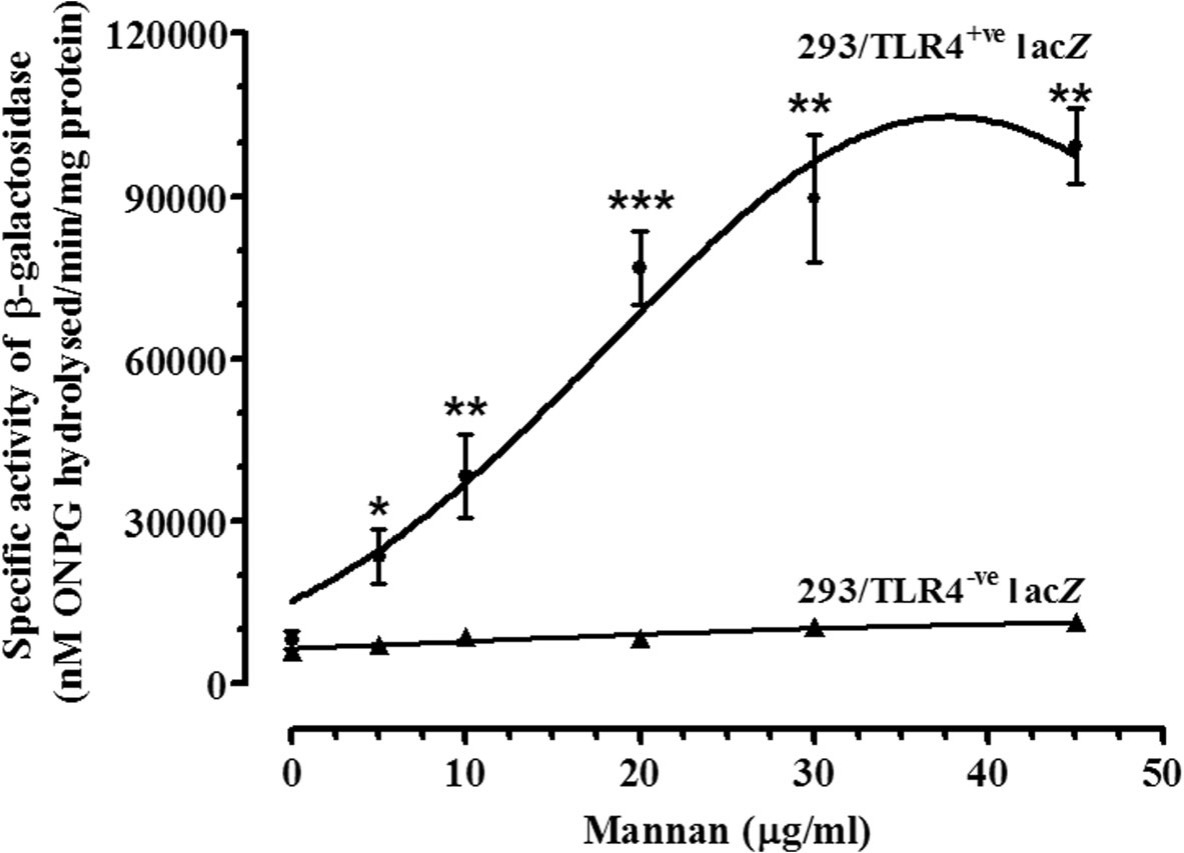
NF-*κ*B Reporter gene assay. Cells were treated with increasing concentration (0–45 μg/ml) of Mannan for 6 h at 37 °C to check the activation of NFκB in terms of specific activity of β-galactosidase as described under materials and methods. Results are expressed as nM of ONPG hydrolysed/min/mg protein ± SD of three independent experiments. Differences with respect to untreated controls were designated significant at values * *p* < 0.05, ** *p* < 0.001 and *** *p* < 0.0001

**Fig. 2:**
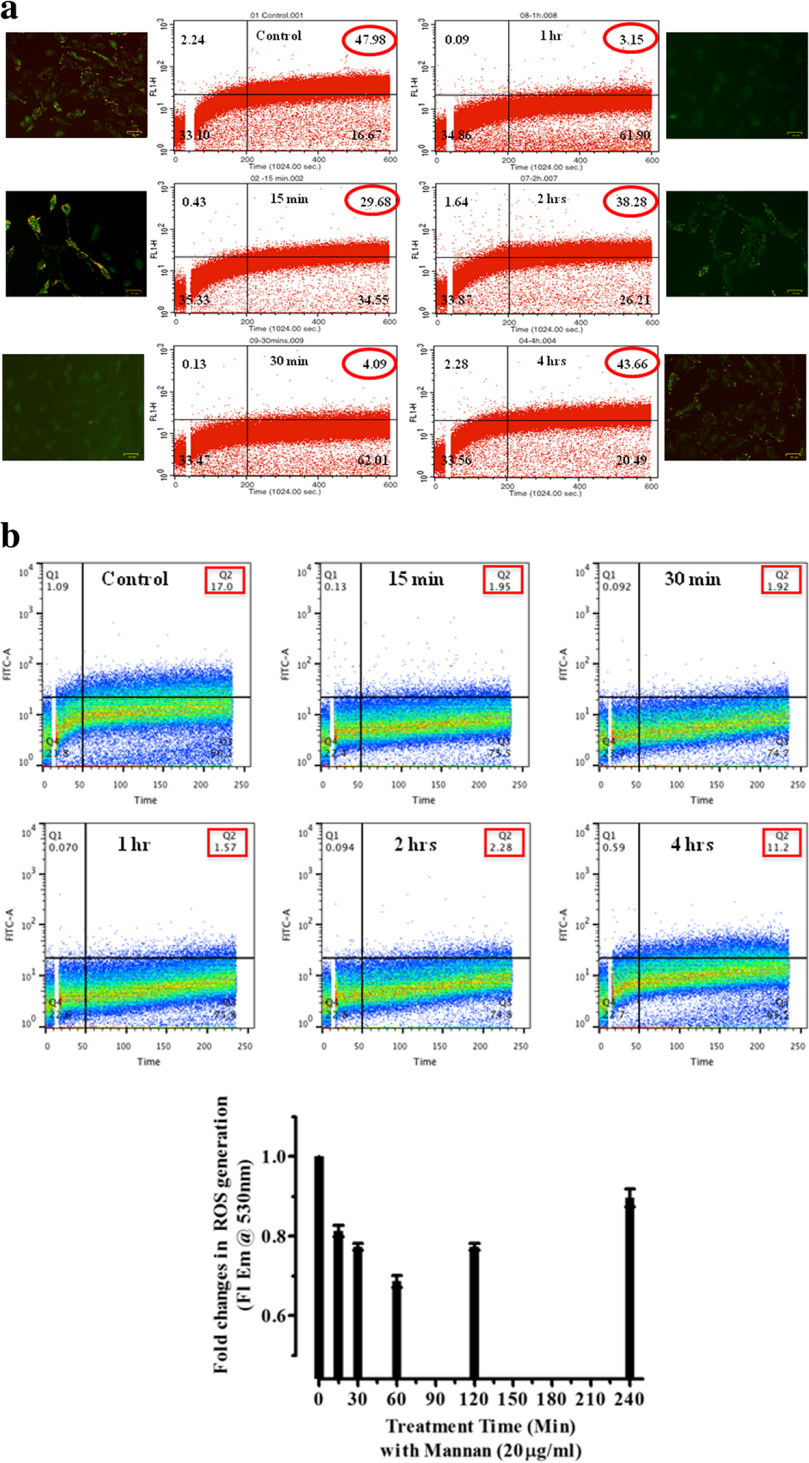
**a**. Time kinetics of DiOC_6_(3) uptake in NKE cells following treatment with Mannan. Mitochondrial Membrane potential mediated sequestration of DiOC_6_(3) in mitochondria of NKE cells was assessed flowcytometrically as described in materials and method section. Briefly, cells were treated with mannan, and dye uptake with time was measured continuously using Ex λ 488 and Em λ 530. The results are representative of three independent experiments. Data was analyzed using FlowJo V10.1 software. Images to assess for changes in membrane potential using JC-1 dye (using green and red channels of ZOE™ Fluorescence microscope) were also acquired after 10 min (incubation with dye) as described in materials and method section. The acquired images were merged and analysed using inbuilt software provided with the microscope. **b**. Time kinetics of ROS generation in NKE cells after treatment with Mannan. ROS mediated oxidation of CM-H_2_DCFDA and measurement of the fluorescent CM-DCF with time as an indicator of redox status of cells. Briefly, cells treated with mannan was harvested and thereafter oxidation of dye was measured flowcytometrically using Ex λ 488 and Em λ 530 as described under materials and methods. Obtained results presented are representative of three independent experiments. Data was analyzed using FlowJo V10.1 software. To confirm the changes in ROS levels following treatment of cells with mannan, the oxidation of CM-H_2_ DCFDA dye was also measured using microplate reader, at Ex λ 488 nm and Em λ 530 nm. Results are representative of mean fluorescence and expressed as fold changes in mean fluorescence

Treatment of cells with mannan showed a concentration dependent increase in activation of NFκB. Mannan (5 μg/ml – 40 μg/ml) showed significant increase in hydrolyzed ONPG conc. (NFκB activity) up to 30 μg/ml, however further increase in concentration showed no significant changes. 293/TLR^-ve^-lac*Z*^*+ve*^ cells were taken as negative control and no significant color development of hydrolyzed ONPG was observed in case of at any treatment concentrations of mannan (Fig. 1). The concentration of mannan in mediating changes in NFκB activation corroborates with changes in intracellular ΔΨm and ROS generation. Changes in fluorescence associated with the uptake of DiOC_6_(3) (cationic lipophilic dye) and JC-1 dyes allows evaluation of alterations in mitochondrial membrane potential in live cells. The time dependent uptake of ΔΨm dependent dye DiOC_6_ (3) was measured by flow-cytometry in NKE cells following treatment with mannan (20 μg/ml). Additionally, formation of ΔΨm dependent aggregates of JC-1 (red) or accumulation of JC-1 (green) was measured microscopically. Cells treated with mannan showed remarkable alteration in ΔΨm with respect to untreated control cells as indicated in upper right quadrant of dot-plots and corresponding image acquired using fluorescence microscope, which was found to be time dependent (Fig. 2a). Maximum decline in ΔΨm (~3% population) was observed at 1 h post-treatment with mannan, which begins to augment with time and returned near to control levels after 4 h of treatment time (~44% of population). The results of changes in ΔΨm using two different dyes and techniques corroborated with the corresponding time interval.

Similarly, the kinetics of oxidation of CM-H_2_DCFDA was also found to be associated with treatment time of cells with mannan (Fig. 2b). Resembling alterations in ΔΨm, reactive oxygen species (ROS) reached its minimum level after 1 h (~2% population in comparison to ~17% of control, upper right quadrant) of treatment with mannan, which was found to increase with time and reached normal level (~13% of population) after 4 h of treatment time. The oxidation of dye measured as mean florescence (fold changes) is indicative of changes in generation of ROS following treatment of cells with mannan at different time intervals and results of both techniques exhibited similar trend and corroborated with the corresponding time intervals (Fig. 2b).

The decrease in both ΔΨm and ROS generation after mannan treatment were found to be transient and their levels were restored by 4 h to those seen before treatment, indicating that the cellular physiology was restored.

### Radio-protective efficacy of mannan in vitro

**Fig. 3:**
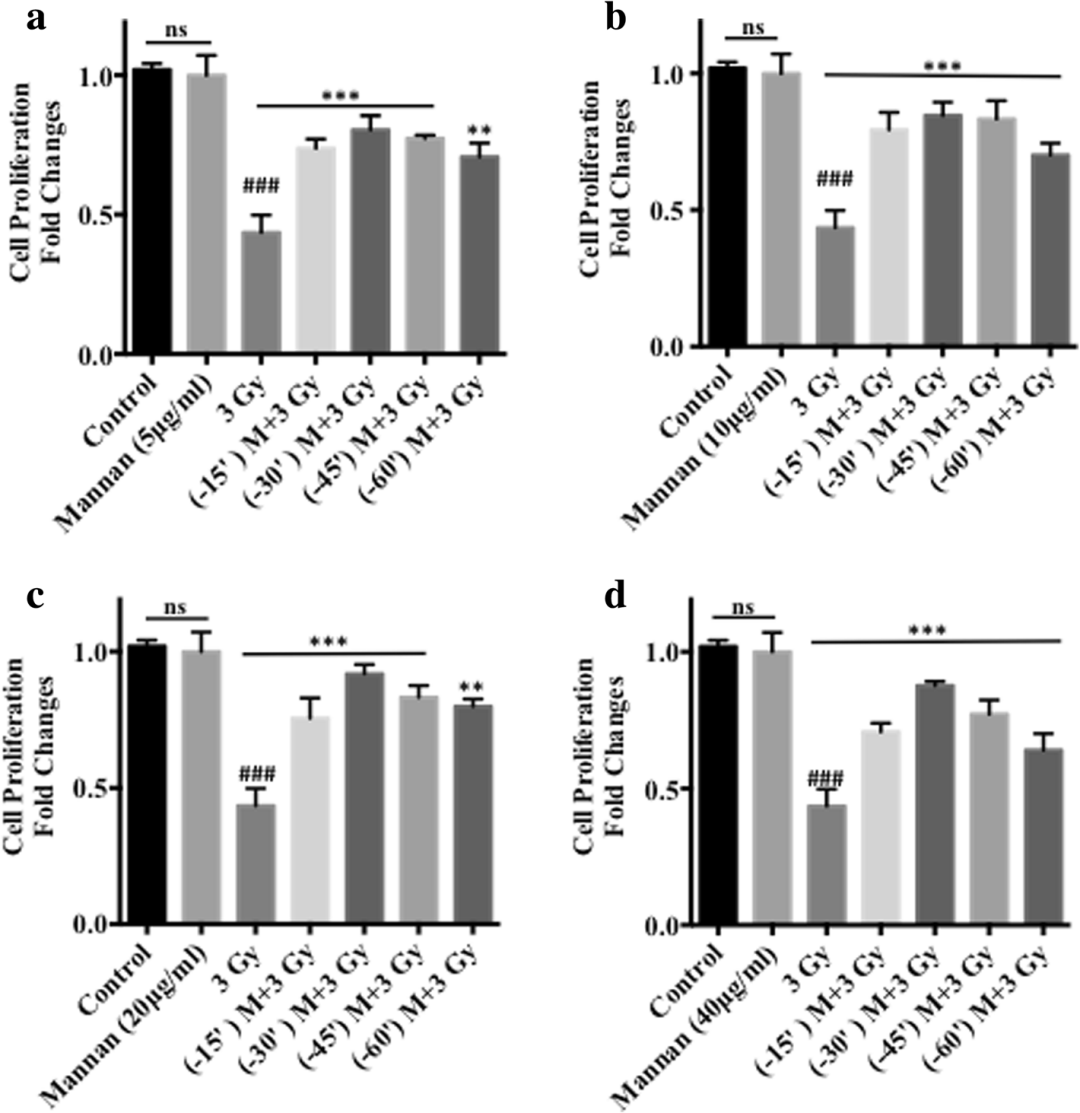
Sulforhodamine B uptake Assay. Effects of pre-irradiation treatment of NKE cells with mannan [(**a**) Mannan conc. 5 μg/ml (**b**) Mannan conc. 10 μg/ml (**c**) Mannan conc. 20 μg/ml (**d**) Mannan conc. 40 μg/ml] for different time intervals was measured in terms of cell proliferation (SRB uptake assay) 96 h post irradiation. Results are expressed as cell proliferation (fold changes) with respect to control ± SD of three independent experiments. Differences were designated significant at values ** *p* < 0.001 and *** *p* < 0.0001 and were labeled as # compared with the sham irradiated control group, * compared with the 3 Gy (radiation only) group

**Fig. 4:**
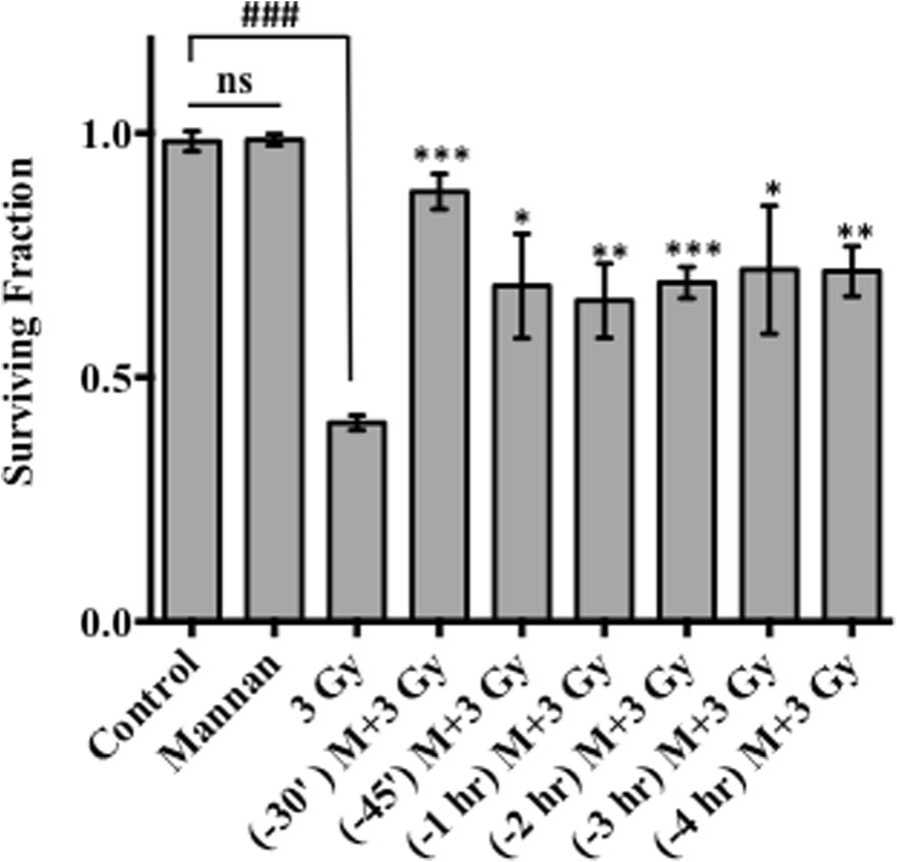
Clonogenic assay. Effect of pre-irradiation treatment of NKE cells with mannan at different time interval was accessed by using colony forming efficiency (CFE) assay as described in materials and method section. After incubation, formed colonies were fixed, stained and counted. Results are expressed as surviving fraction with respect to control ±SD of three independent experiments. Differences were designated significant at values * *p* < 0.05, ** *p* < 0.001 and *** *p* < 0.0001 and were labeled as # compared with the sham irradiated control group, * compared with the radiation group

Cells treated with increasing concentrations of mannan (50 ng/ml - 5 mg/ml) showed no cellular cytotoxicity as measured by SRB uptake assay 96 h post treatment. Moreover, microscopic examination of the cellular morphology following treatment with mannan suggests no significant changes in cell number or cell morphology with respect to control at all tested concentrations (data not shown). Logarithmically growing NKE cells were treated with mannan prior to radiation exposure and thereafter cell proliferation and surviving fraction was evaluated by SRB uptake assay (Fig. 3a-d) and clonogenic assay (Fig. 4) respectively. Cells exposed to 3 Gy gamma radiation exhibited decrease in cell proliferation and surviving fraction as compared to control and mannan alone treated cells. Whereas, cells pretreated with Mannan (5, 10, 20, 40 μg/ml respectively) for different time intervals (15, 30, 45, 60 min) followed by radiation (3 Gy) showed increase in cell survival with respect to irradiated (3 Gy) cells. Maximum survival was observed in cells treated with 20 μg/ml mannan (added 30 min prior to irradiation; Figs. 3 and 4). The results obtained from clonogenic assay substantiate with the cell proliferation data from SRB uptake assay. Treatment of cells with mannan showed no changes in clonogenicity with respect sham irradiated group, however pre-irradiation treatment of cells with mannan enhance survival of cells with respect to the cells exposed to 3 Gy alone.

### Alterations in mitochondrial ETC functions confiscate mannan mediated radiation protection efficacy

**Fig. 5:**
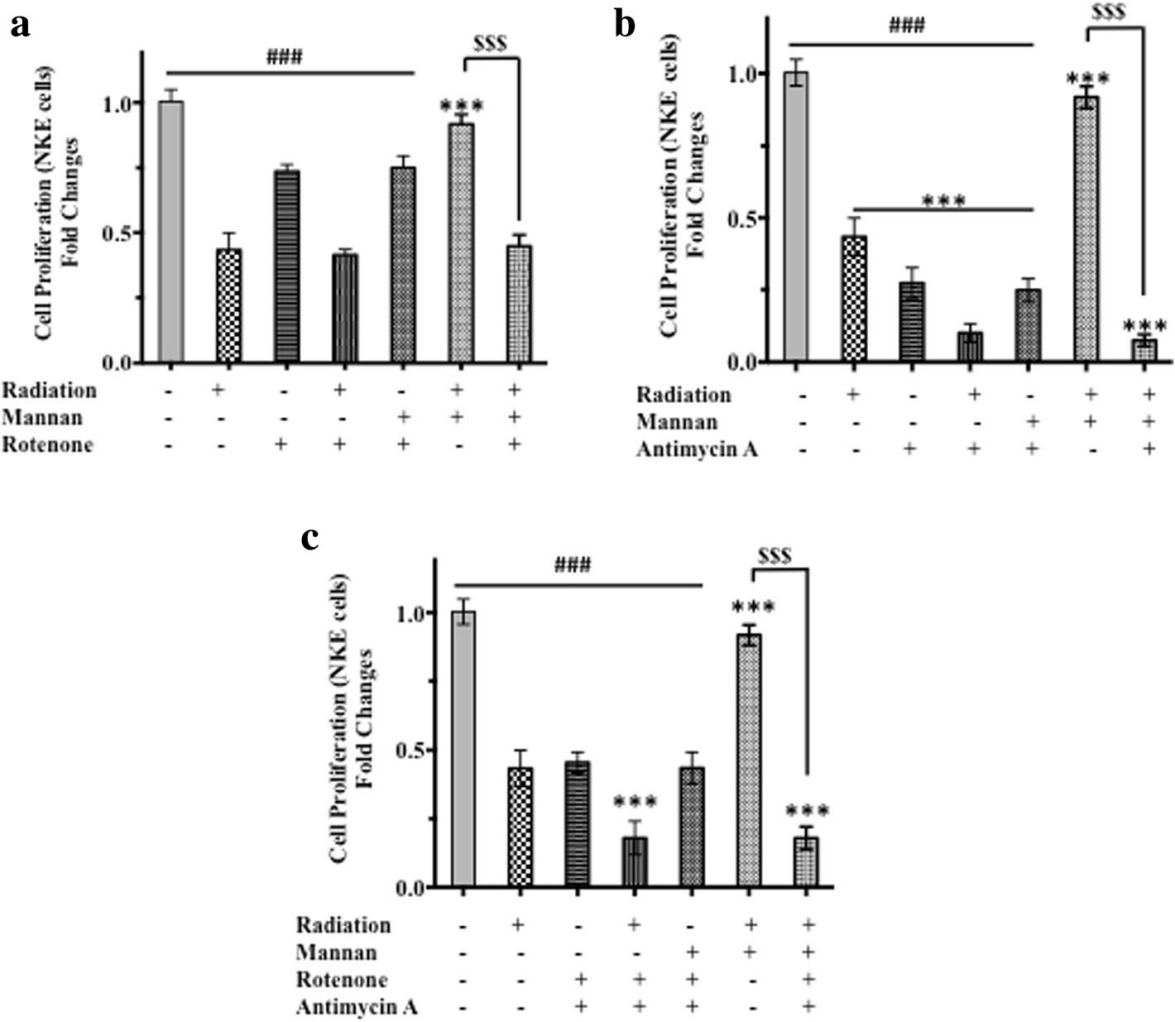
Studies on requirement of functional ETC for radiation protection offered by mannan. The requirement of functionality ETC in radiation protection was assessed using inhibitors of complex I and Complex III. Briefly cells were treated with (**a**) Rotenone, inhibitor of complex I, (**b**) Antimycin A, inhibitor of complex III, (**c**) Both Rotenone and antimycin A, 30 min prior to mannan treatment and/or irradiation followed by SRB uptake after 96 h as described under materials and methods. Results are expressed as fold changes in cell proliferation ± SD of three independent experiments. Differences were designated significant at values *p < 0.05, ** *p* < 0.001 and *** *p* < 0.0001 and were labeled as # compared with the sham irradiated control *group*, * compared with the radiation group, and $ compared with radiation + mannan group

Following irradiation of NKE cells, the fold changes in proliferation was reduced significantly with respect to both control and mannan + radiation group (Fig. 5a-c). Cells treated with rotenone (complex I inhibitor; Fig. 5a) and antimycin A (complex III inhibitor; Fig. 5b) exhibited significant decrease in cell proliferation with respect to control cells (sham treated or mannan treated) as measured by SRB uptake assay (*p* < 0.0001). Cells treated with rotenone followed by irradiation showed further decrease in proliferation with respect to rotenone alone group (*p* < 0.001). Furthermore, cells treated with rotenone followed by mannan treatment showed no changes in proliferation with respect to rotenone alone. In case of combination (rotenone+ mannan followed by radiation exposure) no protection was observed with respect to radiation alone and rotenone+ radiation group (Fig. 5a). Similarly, treatment of cells with antimycin A followed by irradiation found to reduce cell proliferation significantly as compared to cells treated with antimycin A alone (Fig. 5b). Whereas, cells treated with combination (antimycin A + mannan followed by radiation) were found to be similar to antimycin A + radiation group).

Treatment of cells with inhibitors of both complex I and Complex III showed significant decrease in proliferation with respect to cells treated with rotenone alone (Fig. 5c). However, cells with compromised electron transport chain (ETC) functions (treated cells with both rotenone and antimycin A) followed by treatment with mannan did not offer any proliferation advantage with respect to inhibitors treated group (both rotenone and antimycin A). Furthermore in cells with compromised ETC functions (treated with both inhibitors) showed significant decrease in proliferation in case both radiation and cells treated with mannan followed by radiation exposure (Fig. 5c).

### Generation of NKE ρ° cells from NKE cells

**Fig. 6:**
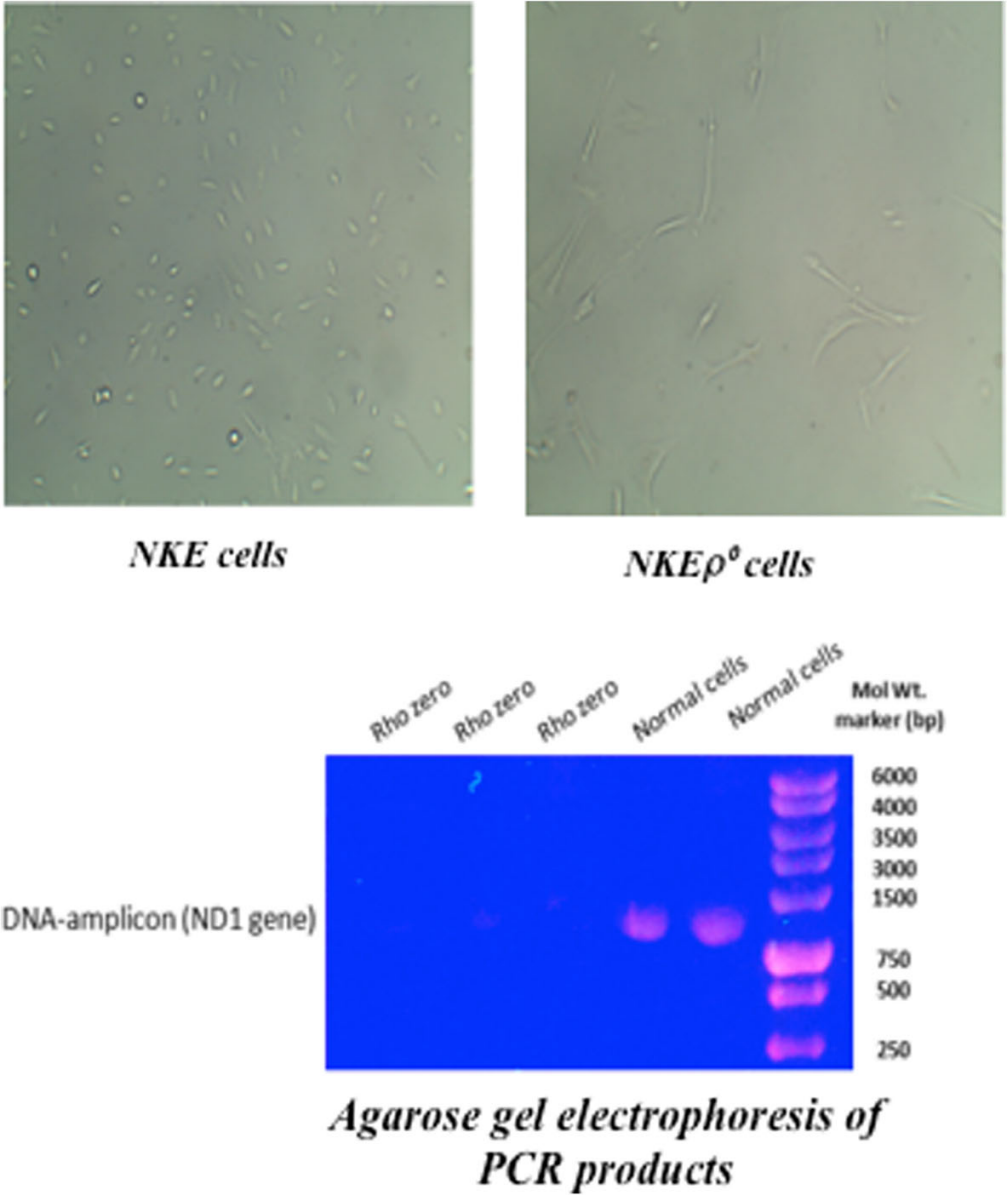
Development of ρ° cells from NKE cells. NKE ρ° cells were generated from NKE cells by selectively depleting mitochondrial DNA using ethidium bromide as described in materials and methods. Mitochondrial DNA was isolated from NKE and ρ° cells, and ND1 gene amplicon from mitochondrial DNA (as amplified using PCR) was checked using agarose gel electrophoresis

Prolonged exposure of NKE cells with ethidium bromide (supplemented with sodium pyruvate and uridine) depleted mitochondrial DNA copy number, and was confirmed by measuring expression of ND1 gene encoded by mitochondrial DNA (Fig. 6). Moreover, NKE ρ°cells exhibits uridine auxotrophy characteristics, which confirm impaired ETC function in these cells.

### Studies on changes in MMP (ΔΨm) and ROS

**Fig. 7:**
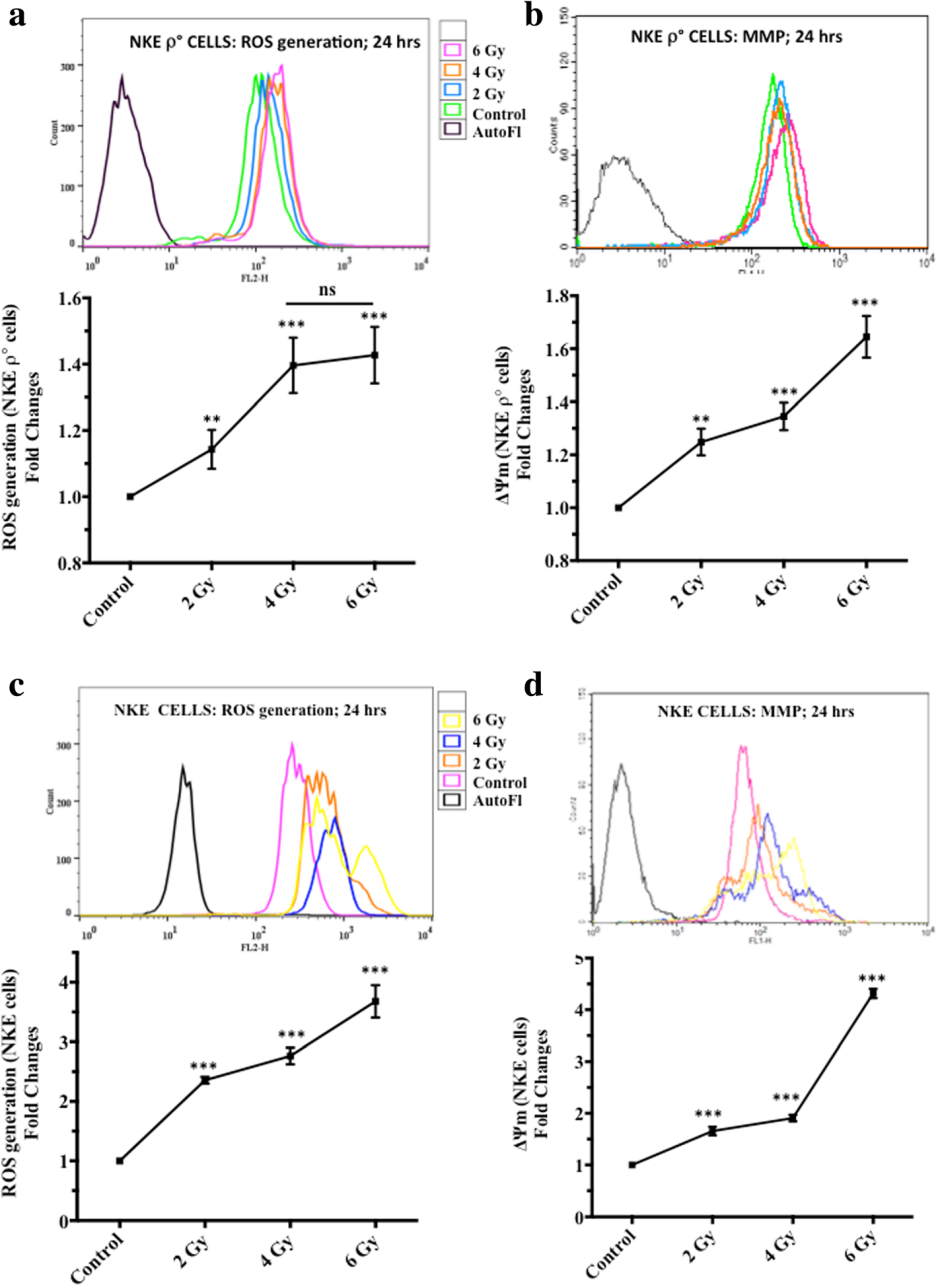
(**a-d**) Changes in ROS generation and ΔΨm. Effect of IR exposure on generation of ROS and changes in ΔΨm in NKE ρ° cells (**a-b**) and NKE cells (**c-d**). ROS levels and ΔΨm were measured by flow cytometry using DHE and DiOC_6_(3), respectively as described in materials and method section. Histograms presented are representative of three independent experiments. Results are also expressed as fluorescence mean ± SD of three independent experiments. Differences were designated significant at values, ** *p* < 0.01, *** *p* < 0.001 and were labeled as * compared with the 3 Gy (radiation only) group. Data was analyzed using FlowJo V10.1 software

The changes in intracellular ROS levels in NKE cells and NKE ρ° cells following increasing dose of γ-radiation (sham, 2 Gy, 4 Gy and 6 Gy) was assessed. Exposure of NKE cells to radiation exhibited dose dependent increase in ROS generation with respect to sham-irradiated control (Fig. 7c). However, radiation induced ROS generation was found to be appreciably lesser in NKE ρ° cells, (1.4 fold of control at 6 Gy irradiation dose, Fig. 7a) with respect to parental NKE cells, (3.5 fold of control at 6 Gy irradiation dose, Fig. 7c).

Similarly, significant increase in ΔΨm was observed following radiation exposure to both NKE and NKE ρ° cells. However the increase in ΔΨm was found to be more prominent and dose dependent in NKE cells (4.3 fold of control at 6 Gy irradiation dose, Fig. 7d) with respect to NKE ρ° cells (1.6 fold of control at 6 Gy irradiation dose, Fig. 7b).

### Studies on NKE ρ° cells, TLR null cells and transformed cells

**Fig. 8:**
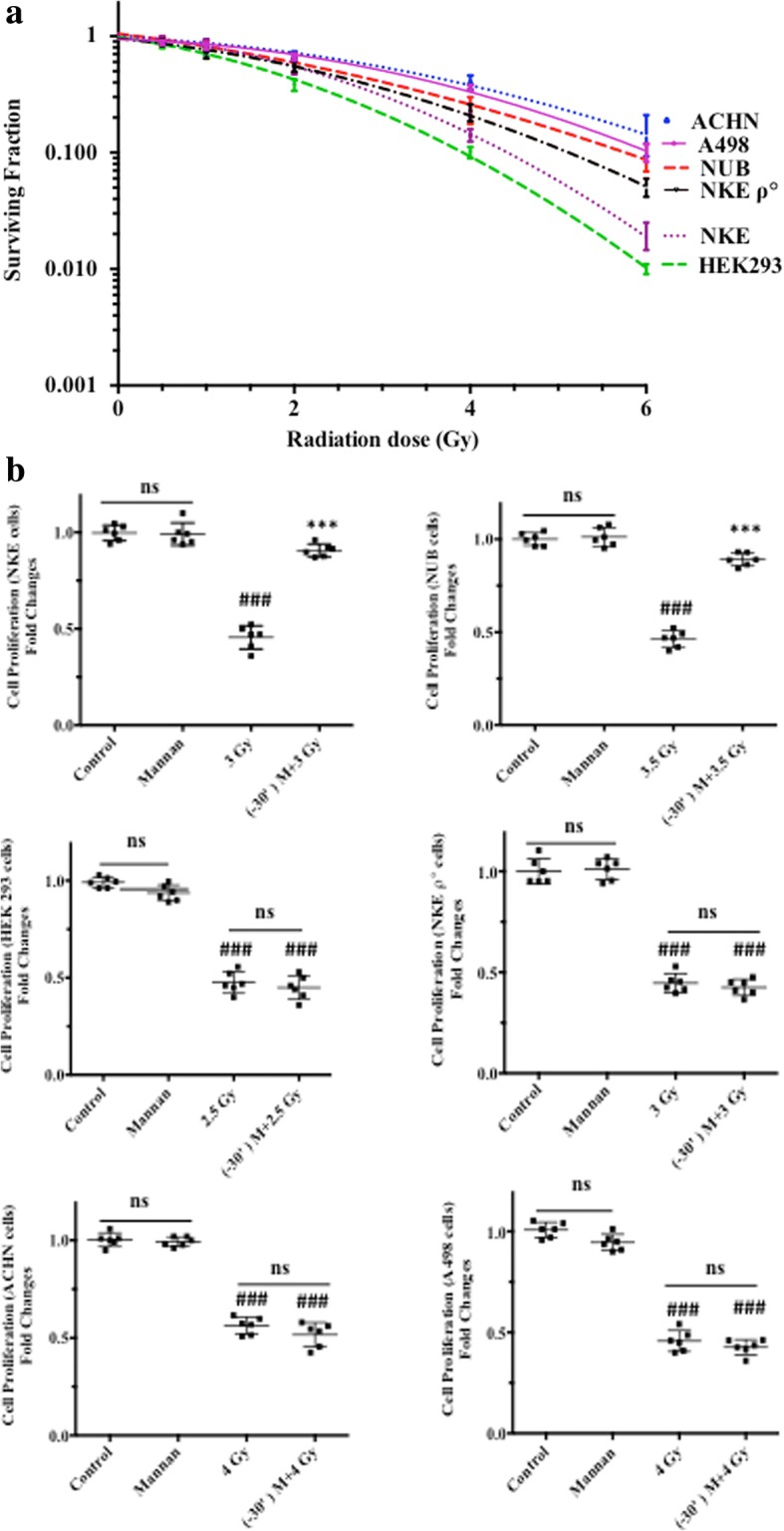
(**a**) Clonogenic assay. Radiation-mediated changes in the percentage survival of cells. Cells were exposed to increasing doses of radiation and clonogenic efficacy of NKE cells, NUB cells, HEK 293 cells, NKE ρ° cells, ACHN cells, A498 cells was measured as described in the methods section. After incubation visible colonies were counted and surviving fraction was calculated with respect to control. Results are expressed as the percentage of cells surviving after treatment with respect to the control ±SD of three independent experiments. *p* < 0.05 was considered as level of significance. (**b**) Sulforhodamine B uptake Assay. Effect of pre-irradiation treatment of mannan on cells [NKE cells, NUB cells, HEK 293 cells, NKE ρ° cells, ACHN cells, A498 cells] was measured by using SRB uptake assay 96 h post radiation exposure (as described in materials and methods section). Results are expressed as cell proliferation (fold changes) with respect to control ±SD of three independent experiments. Differences were designated significant at values, ***p < 0.001 and were labeled as # compared with the sham irradiated control group, * compared with the 3 Gy (radiation only) group

Cell survival and colony forming efficacy (CFE) of NKE cells, NUB cells, HEK 293 cells, NKE ρ° cells, ACHN cells, A498 cells was measured. Exposure of cells to gamma radiation influenced CFE of cells in dose dependent manner. Radiation dose (~LD_50_%) used to assess radio-protective efficacy of mannan was based on respective cellular radiation sensitivity (Fig. 8a). Cells of different nature (normal, NKE ρ°, TLR null and transformed) were used to assess radio-protective efficacy of mannan in normal vs transformed cells and requirement of TLR and functional electron transport chain (Fig. 8b). Treatment of cells with mannan alone was found to be nontoxic and no significant changes were observed in relation to proliferation. Mannan pretreatment in normal cells (NKE and NUB cells) showed significant increase in cell survival when compared with corresponding irradiated cells (96 h; SRB uptake). NKE ρ° cells and HEK293 cells were utilized to understand role of functional ETC and TLR function respectively, in mannan-mediated modification of radiation response in normal cells. NKE ρ° cells and HEK293 cells exposed to radiation exhibited significant decrease in proliferation as compared to control and mannan alone treated cells. Mannan treatment prior to IR exposure did not offer radiation-protection to NKE ρ° cells (deficient in ETC functions) as well as to HEK293 cells (TLR null cell line) indicating the importance of TLR function as well as functional ETC for radiation protection pathway involved. Furthermore, mannan pretreatment did not offer any survival advantage in transformed cells (ACHN and A498).

### Mannan pretreatment increases expression of SOD I (cu-Zn SOD), SOD II (MnSOD) in both NKE parental as well as NKE ρ° cells

**Fig. 9:**
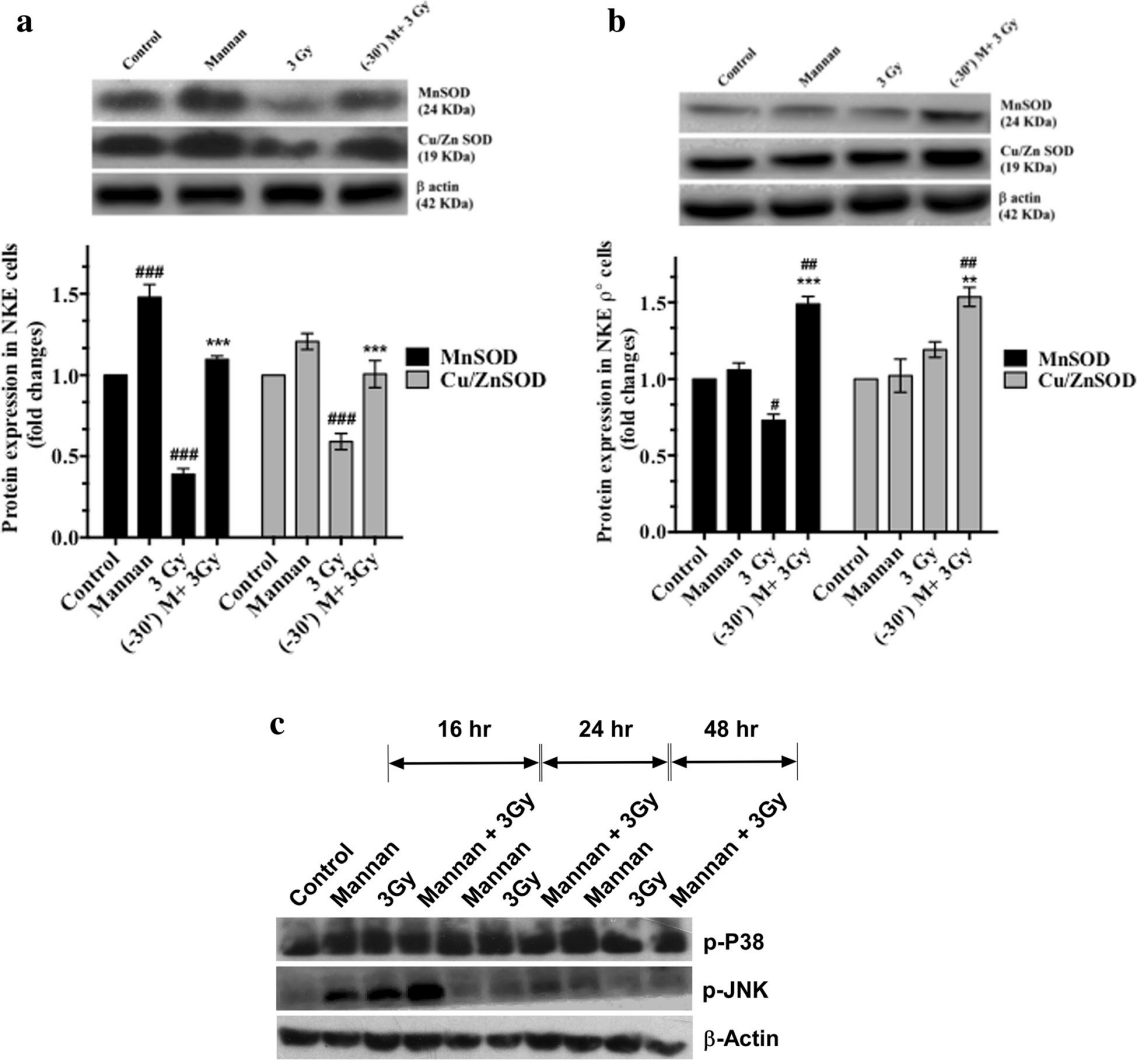
(**a-b**) Expression of Mn SOD and Cu/Zn SOD in NKE and NKE ρ° cells. Cells were treated, harvested and samples were resolved on 8–20% gradient SDS-polyacrylamide gel followed by transfer on to PVDF membrane and blotted using specific antibodies as described in materials and method section. β-actin was used as a loading control. Results are expressed as Protein expression (fold changes) with respect to control ±SD. Differences were designated significant at values, *** *p* < 0.001 and were labeled as # compared with the sham irradiated control group, * compared with the 3 Gy (radiation only) group. (**c**) Phosphorylation of JNK and P38. To assess role of stress response pathways in mannan mediated protection activation of JNM and P38 was measured as phosphorylation status. Briefly, cells were harvested, and equal quantity of cellular proteins were resolved on 8–20% gradient SDS ployacrylamide gel, followed by transfer on to PVDF membrane and blotted using corresponding primary and secondary antibodies as described in materials and methods section. The changes in phosphorylation were compared with radiation and sham irradiation groups at different time intervals. β-actin was used as a loading control

Superoxide dismutase (SOD) enzymes are critical intrinsic antioxidants, which neutralize superoxide radicals in both cytosol and mitochondria of cells, thereby maintaining cellular redox balance. NKE cells treated with mannan alone showed significant increase in levels of both SOD I and SOD II. Moreover, the expression of both SOD I and SOD II were found to be higher in case of mannan + 3Gy group at 24 h post irradiation in both NKE as well as NKE ρ° cells with respect to their respective irradiated (3Gy) group (Fig. 9a and b).

### Assessment of phosphorylation of JNK and p38

Stress response pathways are mediated through external or internal stimuli and are known to be crucial in relation to determination of cell survival. Phosphorylation of both p38 and JNK could be due to environmental changes, DNA damages, growth factors radiation exposure etc. The levels of change may be indicative of activation of cells against stress conditions. Treatment of cells with mannan increased phosphorylation of JNK and p38 upto 16 h and thereafter it was restored to normal or untreated control (Fig. 9c). Exposure of NKE cells to 3 Gy radiation also increased phosphorylation upto 24 h with respect to unirradiated cells. In case of mannan + 3 Gy group higher levels of p-JNK was observed with respect to control and 3 Gy alone, though there was no change in case of p-p38 levels.

### Mannan pretreatment in NKE cells minimizes oxidative modification of mitochondrial membrane phospholipids instigated by IR exposure

**Fig. 10:**
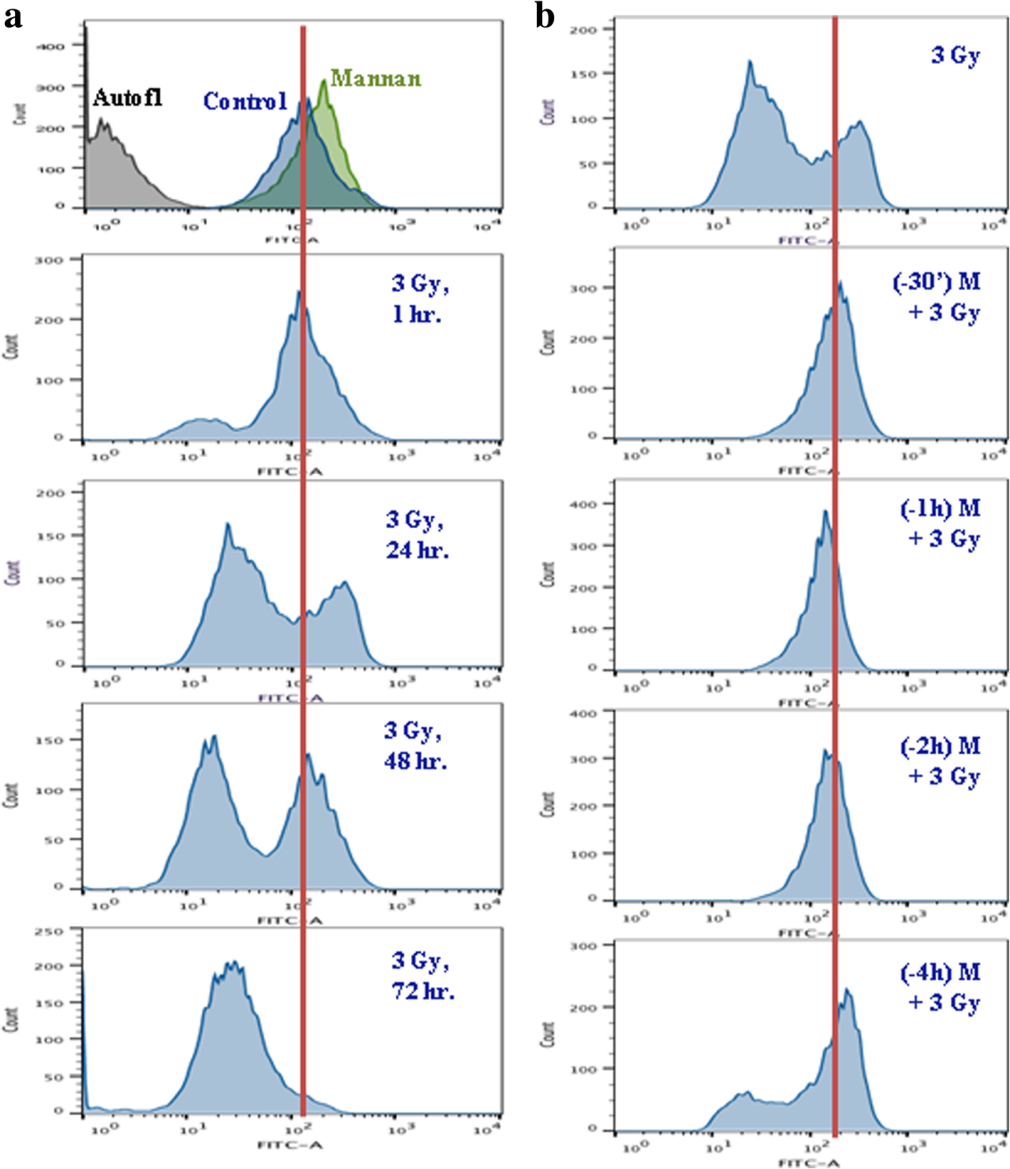
(**a-b**) IR induced changes in oxidative modification of mitochondrial phospholipids (Cardiolipins). Effect of pre-irradiation treatment with mannan on NAO incorporation was measured to assess oxidative modification of cardiolipins as described under materials and methods section. (**a**) Radiation-induced (3 Gy) oxidation of Mitochondrial Phospholipids measured at different time intervals. (**b**) Effects of mannan pretreatment on radiation induced peroxidation of cardiolipin at 24 h post irradiation. Fluorescence of NAO was acquired flow cytometrically at Ex λ 488 nm and Em λ 530 nm. The data were analyzed using FlowJo V10.1 software. The data presented is representative of findings from three independent experiments

10-nonyl acridine orange (NAO) is a fluorescent dye that interacts selectively to reduced cardiolipins, a molecule that is localized in the inner mitochondrial membrane [26]. As measured by NAO incorporation, alteration in mitochondrial cardiolipin content was studied at different time intervals following exposure of cells to 3Gy radiation. NKE cells exposed to 3Gy dose showed decrease in incorporation of NAO with increase of time, depicted as shift of histogram towards left with respect to control, which indicates oxidative modification of lipids with time (Fig. 10a).

Cells treated with mannan showed no significant changes in NAO incorporation. However, treatment of cells with mannan (up to 2 h) followed 3 Gy irradiation found to protect cells in relation to radiation-mediated modification of cardioplins with respect to radiation alone (3 Gy) group (Fig. 10b). The cells treated with mannan up to 4 h followed by 3 Gy radiation exposure showed decrease in protection efficacy as histograms started shifting towards left.

## Discussion

Toll-like receptors are pattern recognition receptors with the ability to detect initial danger signal against microbial infections. Since most TLR ligand(s) are known to activate NFκB pathway and alter mitochondrial function [5, 11, 12], we first intend to identify the concentration specific range of mannan that can activate NFκB pathway. As expected mannan was found to increase activation of NFκB at all concentration studied (Fig. 1). However, trend of increased activation was found to be saturated beyond 30 μg/ml concentration of mannan.

Apart from concentration and time dependent activation of NFκB, we have also identified mannan-mediated alterations in both ΔΨm and ROS in NKE cells. Moreover, our recent studies also suggested that pre-irradiation treatment of cells with mannan restored mitochondrial dynamics and ETC function, which might also have contributed in radiation protection of cells [25]. Mitochondrial ETC generates potential gradient across the inner mitochondrial membrane along with ROS as a by-product, which gets neutralized by intrinsic antioxidants *viz*. SOD I and SOD II, catalase etc. The abrupt decrease in ΔΨm and endogenous ROS levels following treatment of cells with mannan suggests possible alterations in mitochondrial fusion/fission or ETC expression and functioning [1, 27]. Interestingly, this decrease in both ΔΨm and ROS was found to be transient and normal levels were restored within few hour of treatment (Fig. 2a and b). Contrarily, activation of TLRs is known to augment ROS [12]. Since, mitochondria is known to regulates various processes *viz.* OXPHOS, apoptosis etc. and plays crucial role in cell signaling, we envisioned to utilize phenomenon of perturbation of ΔΨm and ROS to study biological effects of radiation following treatment of cells with mannan. To our expectations, NKE cells pre-treated with mannan and exposed to radiation showed enhanced survival (Figs. 3 and 4), as radiation is known to alter ΔΨm and ROS levels and thereby cause damage to cellular biomolecules [28]. To assess role of mitochondrial ETC in radiation protection offered by mannan, cells were treated with inhibitors of ETC complexes *viz.* rotenone and antimycin A. The radiation protection ability to cells offered by mannan was completely abolished when cells were treated with inhibitors (Fig. 5a-c). Moreover, antimycin A was found to sensitize cells, when exposed to radiation, which could be due to increased leakage of electrons from ETC components. The inhibitors used to inhibit ETC function were toxic enough to derive useful survival information, and showed toxicity following radiation exposure. Therefore, we developed NKE ρ° cells from NKE cells (Fig. 6) and utilized them to understand necessity of functional ETC in mannan-mediated modification of radiation response(s).

TLR expressing normal cells (NKE and NUB), rho zero cells (NKE ρ°), transformed cells (ACHN and A498) and TLR-null cells (HEK293) were treated with mannan followed by irradiation depending on radiation sensitivity of cells. Interestingly, mannan treatment prior to radiation exposure offered significant survival advantage to TLR expressing normal cells (NKE and NUB cells) but not to NKE ρ° cells, TLR null cells, and transformed cells exposed to γ-radiation (Fig. 8b). Previously, mannan (from *A. saponaria*) has been shown to inhibit tumor cell proliferation and activation in vitro in several tumor cell lines [24]. Mannan (from *Saccharomyces cerevisiae*) exhibits strong growth-inhibitory activity in vivo against mouse-implanted Sarcoma 180 and Ehrlich-carcinoma solid tumor [23]. Regulation of glycolytic pathway and oxidative pathway varies in transformed cells depending on various factors including malignancy of tumor. Most transformed cells are known to exhibit high glycolytic rate and significantly less oxidative metabolism [29]. ACHN is sensitive towards glucose deprivation [30], suggesting its more dependence on glycolytic pathway than oxidative phosphorylation (OXPHOS). Since modification of radiation response by mannan is dependent on ETC functions, less OXPHOS in ACHN cells might be one of the reasons that mannan pretreatment did not offer any survival advantage against radiation exposure. TLR null cells (HEK293) on the other hand are not able to initiate TLR mediated signaling pathways because of absence of imperative TLRs, which could be possible reason of no protection against radiation.

ROS are important components in a number of mitochondria-initiated signaling pathways [31, 32]. Several studies have indicated that both molecular and enzymatic antioxidants play a critical role in reducing ill effects of radiation [33, 34]. It has been established that SOD plays a central role in protecting cells against ROS induced injury during radiation exposure [1]. Both Cu/Zn SOD and MnSOD are the major ROS detoxifying enzyme of cytosol and mitochondria respectively and overexpression of MnSOD has shown to be able to protect cells from the damage induced by radiation induced ROS [5]. In the present study, it was found that pre-irradiation treatment of normal as well as NKE ρ° cells with Mannan was found to increase endogenous levels of both Cu/Zn SOD and MnSOD when compared with control or irradiated cells. Exposure of cells to radiation at the time of maximum alterations in mitochondria functioning and increased levels of SODs (induced by mannan) might also be responsible in reducing ill effects of ionizing radiation in both NKE and NUB (normal TLR expressing) cells. On the contrary, cells bearing impaired ETC (NKE ρ°), showed increased SOD expression 24 h post irradiation in mannan pretreated cells, however no protection was observed against radiation in terms of cells proliferation indicating necessity of functional ETC. NKE ρ° cells lack critical respiratory chain catalytic subunits encoded by the mitochondrial genome and are deficient in OXPHOS [31]. Since NKE ρ° cells themselves are deficient in ETC functions; therefore, unlike their parental counterparts, treatment of these cells with radiation showed significantly less changes in ROS generation (Fig. 7a) and ΔΨm (Fig. 7b) in comparison to NKE cells (Fig. 7c-d).

Mitochondria are vulnerable to oxidants because they are the major source of free radicals and Fenton catalysts in the cells and are limited in their ability to cope with oxidative stress. Reactive oxygen species (ROS) acts as mediator of ionizing radiation-induced cellular damage [27, 33]. Mitochondrial inner membrane is rich in phospholipids, which are essential for several vital processes taking place inside mitochondria including ETC. Cardiolipin are important membrane phospholipids, primarily present in inner mitochondrial membrane. Membrane phospholipids are at increased risk of peroxidation by ROS continuously produced in the vicinity, which increases to several folds on exposure to radiation. Due to membrane fluidity, ROS can freely permeate free energy barrier, and access the potential oxidation sites (both lipids and proteins) along the membrane [35]. Oxidative damage to membrane phospholipids may result in structural defects of the phospholipid bilayer by altering membrane fluidity and ion permeability leading to destabilization of the membrane structure and possibly to membrane breakdown. These events may be associated with compromised function of mitochondrial ETC and may lead to reduced OXPHOS efficiency or cell death [36, 37]. Furthermore, destabilization of the bilayer might also favor ROS penetration and promote overspreading of the oxidative attack to other membrane components in the cell [35]. NAO specifically binds cardiolipin moieties inside inner mitochondrial membrane [26]. Decrease in NAO fluorescence in cells exposed to radiation corresponds to oxidation of cardiolipin moieties, since NAO cannot bind oxidized cardiolipin and restoration of florescence in mannan-pretreated cells suggests that mannan pretreatment prevented peroxidation of cardiolipins (Fig. 10b). On radiation exposure, membrane phospholipids are at increased risk of peroxidation by ROS and can lead to oxidation of cardiolipin inside mitochondrial inner membrane, resulting in amplification of ROS and changes in ΔΨm (Fig. 7c-d and 10a). Mannan pretreatment increases SOD levels in cells and mitochondria, which could have also been associated with its radiation protection efficacy. Moreover, mannan has earlier been shown to possess antioxidant properties, in vitro [22]. All these factors might have synergistically contributed to prevent peroxidation of cardiolipin inside mitochondrial inner membrane. It has been shown that direct communication between TLRs and mitochondria occurs inside cell and a novel pathway has been elucidated that links innate immune signaling to mitochondria [12]. Mannan is a bulky oligosaccharide that cannot enter cell (unpublished data), and we are currently trying to identify how signal(s) reaches to mitochondria when membrane bound TLR interact with mannan.

As a part of innate immune response, TLR agonists are known to activate stress responsive and NF κB pathways, to eliminate or reduce the biological effects caused by microbes/ microbes associated molecular patterns (MAMP/PAMP). Activation of MAPK is known in case of when cells treated with TLR agonists [5, 9, 11]. In the presents investigation activation or phosphorylation of both P38 and JNK could be due to TLR-Mannan mediated stimulus, which exhibited time dependent decrease. The levels of p-JNK was found to be significantly higher with respect to both control and Radiation alone upto 24 h (Fig. 9c). Pre-activation of JNK by mannan treatment might have stimulated cells to prepare against radiation induced stress resulting in increased survival, as observed in case of combination groups. However, the detailed mechanism related to involvement of stress responsive pathways still needs to be unraveled.

Mannan oligosaccharide (MOS) is largely non-toxic when taken orally; it supports the gut microflora, increases microvilli surface area and goblet cell numbers in small intestine of mannan-supplemented animals [13, 20]. Moreover, All TLRs have been reported to express in the small intestine in humans and mice [38]. Results from our previous studies have confirmed that mannan does not get absorbed through gut, when taken though oral route [39] suggesting its residence (even in small quantities) is sufficiently long enough in gut to initiate imperative TLR signaling and other protective effects in mice. Since, it stimulates epithelial barrier structure and functionality of intestinal mucosa; it has been tested against radiation induced gastro-intestinal acute radiation syndrome (GI-ARS), in our laboratory (A detailed report of the in vivo study will be communicated elsewhere). However, validation of radio-protective potential in other animal models including, nonhuman primates, is important for interpreting MOS as a radiation countermeasure agent.

## Conclusion

**Fig. 11:**
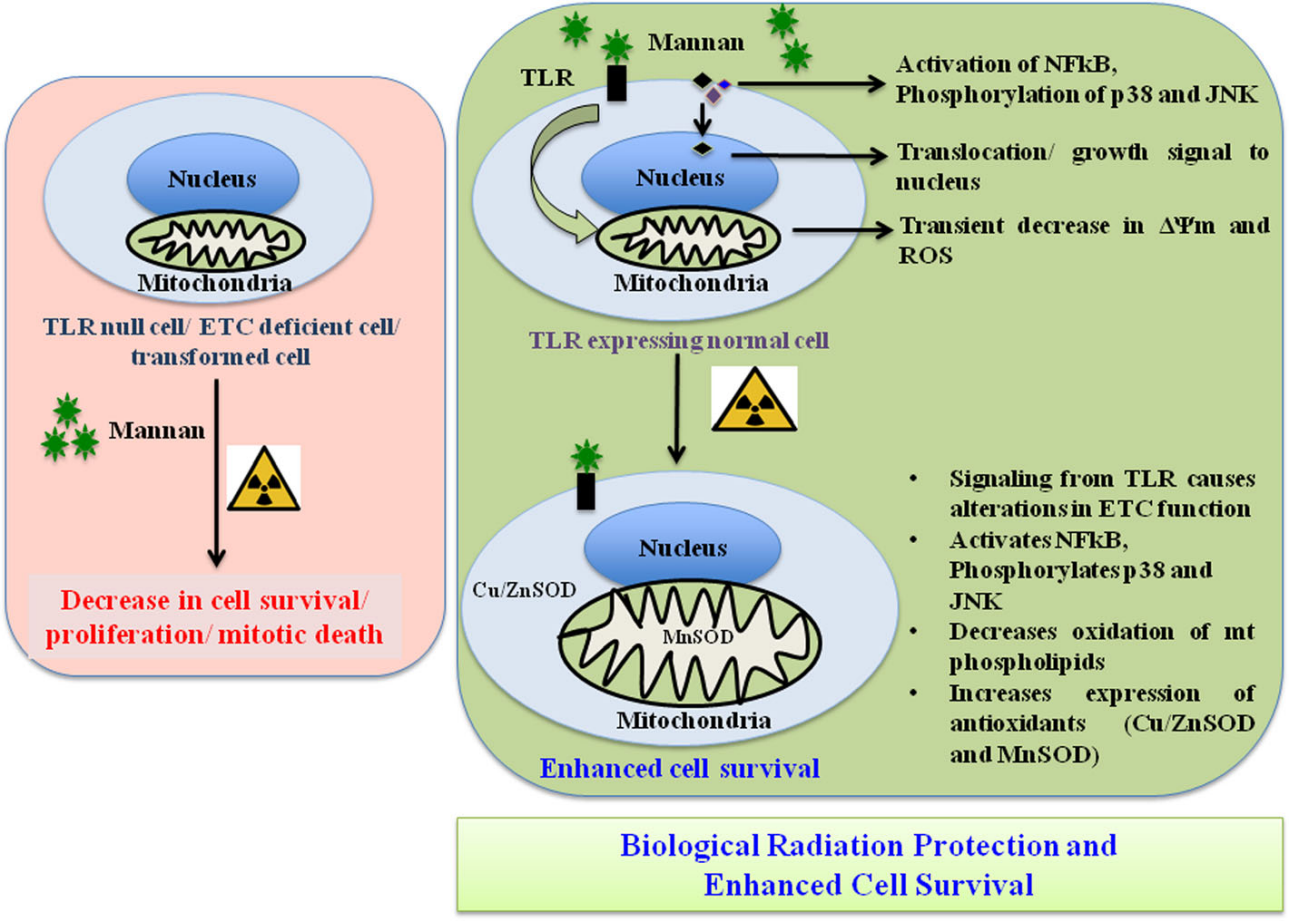
Summary of mechanism of action of MOS. Cells treated with or without MOS showed altered MMP and Enhanced ROS generation. Pre-irradiation treatment of cells with MOS specifically protected normal cells, suggesting requirement of TLR and functional integrity of ETC. MOS also found to upregulate MnSOD and Cu/ZnSOD expression and activated NFκB. All these factors might have collectively contributed towards radio-protective efficacy unveiled by MOS

Exposure of cells to lethal doses of radiation, at a specific stage of perturbed mitochondrial membrane potential and ROS offers significant survival advantage against radiation exposure, in TLR expressing normal cells. TLR and mitochondrial ETC functions are inevitable in radio-protective efficacy exhibited by mannan. Under in vitro system, mannan counters radiation response through activation of NFκB, p38 and JNK, alteration in mitochondrial physiology, increase in MnSOD and Cu/ZnSOD expression, and decrease in peroxidation of cardiolipin inside mitochondrial inner membrane. All these factors might have collectively contributed towards radio-protective efficacy unveiled by mannan that ensures cell survival (Fig. 11).

## Methods

### Chemicals

All chemicals used in study were of analytical grade and were either procured from Indian manufacturer (SRL India, HiMedia chemicals) or obtained from Sigma Aldrich (St Louis, MO), Thermo Fisher Scientific Inc. (USA) etc. Roswell Park Memorial Institute (RPMI-1640) medium, High glucose Dulbecco’s Modified Eagle’s medium (DMEM), Eagle’s Minimum Essential Medium (EMEM), penicillin, streptomycin, blasticidin, trypsin, mannan, rotenone, antimycin A, bovine serum albumin (BSA), ethidium bromide, sodium pyruvate, uridine, protease and phosphatase inhibitor cocktails, REDTaq ReadyMix PCR reaction mix, antibodies (anti MnSOD, anti-SOD1, phospho JNK, phospho p38, anti rabbit/ mouse-HRP, anti β-actin-HRP) etc. were obtained from either Sigma Aldrich (St Louis, MO) or Cell Signaling technology, USA, whereas 3, 3’-DihexyloxacarbocyanineIodide [DiOC_6_ (3)], JC-1, 5-(and-6)-chlormethyl 2′,7’dichlorodihydrofluorescein diacetate acetyl ester [CM-H_2_DCFDA], 10-N-nony1–3, 6-bis [dimethylamino] acridine (NAO), Dihydroethidine (DHE), sulphorhodamine-B (SRB), fetal bovine serum (FBS), Keratinocyte-SFM (1X) were procured from Thermo Fisher Scientific Inc. (USA).

### Cell culture

Human normal kidney epithelial (NKE) cells and human normal urinary bladder cells (NUB) were received as kind gift from Dr. Andrei V. Gudkov, Roswell Park Cancer Institute, Buffalo, USA. ACHN, A498, and HEK 293 cells were obtained from National Centre for Cell Sciences, Pune, India. Normal kidney epithelial rho zero (NKE ρ°) cells were generated from NKE cells by selectively depleting mitochondrial DNA by prolonged exposure to ethidium bromide. 293/hTLR4 cells (HEK 293 cells stably transfected with the human TLR4 gene) were obtained from InvivoGen (San Diego, USA) and were transfected with Lenti NFκB reporter conjugated with Lac*Z* (SA Biosciences, Frederick, MD, USA). NKE parental and ρ° cells were maintained in RPMI-1640; ACHN and A498 in EMEM, HEK293 cells in High glucose DMEM medium. RPMI 1640, EMEM and high glucose DMEM media were supplemented with 10% (*v*/v) heat-inactivated FBS, 1% non essential amino acids, 100 units/ml of penicillin and 100 μg/ml of streptomycin, pH 7.4 to maintain optimal growth of cells at 37 °C in humidified atmosphere of 5% CO_2_. NKE ρ° cells were specifically maintained as indicated with additional supplementation with 100 μg/ml pyruvate and 50 μg/ml uridine. NUB cells were maintained in antibiotic free Keratinocyte-SFM (1X) media supplemented with human recombinant Epidermal Growth Factor 1–53 (EGF 1–53) and Bovine Pituitary extract. All the cell lines were examined for mycoplasma contamination and experiments were performed on exponentially growing cells and were subcultured twice a week.

### Preparation of Mannan solution

Mannan was dissolved (20 mg/ml stock) in sterile PBS (1X) under aseptic conditions. Treatments of cells with mannan were performed as per indicated concentration(s), and time.

### Gamma irradiation of cells

Irradiation was done using Bhabhatron- II Telecobalt unit (Bhabha Atomic Research Center, Mumbai, India) at dose rate 2.25–2.55 Gy/min. Radiation dosimetry of unit was carried out by certified radiation safety officer in the institute and Baldwin Farmer secondary dosimeter and Fricke’s chemical dosimeter methodologies were used [2]. Briefly, logarithmically growing cells were seeded as per experimental design (for clonogenic assay 200 cells/ 60 mm cell culture dish, SRB assay ~2500–3000 cells/well in 96 well culture plate etc.) followed by various treatments. Changes in biological effects of radiation in cells were studied thereafter as mentioned in different sections of methodologies.

### Treatment protocol

NKE cells were treated with increasing concentrations of mannan (100 ng/ml to 5 mg/ml) to perform toxicity studies. For other studies, cells were divided into four groups: Control (Sham treatment), mannan alone, radiation alone, and combination (mannan + radiation). For all experiments 20 μg/ml concentration of mannan was used, 30 min prior to IR exposure, unless otherwise stated. Rotenone and antimycin A was added to cells at 50 nM and 1 μM concentrations respectively, 1 h prior to radiation exposure or/and Mannan pretreatment.

### Reporter gene assay

293/hTLR4 and 293/TLR null cells were transfected with Lenti NFκB reporter conjugated with LacZ (SA Biosciences, Frederick, MD, USA) by using escort reagent (Sigma Aldrich, St. Louis, MO) according to manufacturer’s protocol. Stable clones were obtained using puromycin selection (1 μg/ml). Reporter assay was performed as described by *Adhikari* et al. [40] with minor modifications. Briefly, cells were treated with increasing concentrations of mannan for 6 h at 37 °C to check the activation of NFκB. After incubation media was replaced and attached cells were washed gently with phosphate buffered saline. Lysis buffer [10 μl; 0.25 M tris (2-amino-2-hydroxymethyl- propane-1,3-diol), pH 8.0] was added in each well, followed by three times freeze thaw cycles of 5 min each. Cleavage buffer [50 μl; (0.6 M Na_2_HPO_4_.7H_2_O, 0.4 M NaH_2_PO_4_.H_2_O, 0.1 M KCl, and 0.01 M MgSO_4_.7H_2_O, pH 7) with 0.135 μl β-mercaptoethanol and 17 μl ortho-nitro-phenyl- β-galactoside (ONPG, 4 mg/ml)] was added per well. Plate was incubated at 37 °C till a light yellow colour develop and incubation time was recorded. 125 μl stop buffer (1 M Na_2_CO_3_) was added per well and absorbance was measured at 420 nm. The amount of ONPG hydrolysed was calculated using the following formula:

$$ \mathrm{nMoles}\ \mathrm{of}\ \mathrm{ONPG}\ \mathrm{hydrolyzed}=\left[{\mathrm{OD}}_{420}\right]\ \left[1.92\ast {10}^5\mathrm{nl}\right]/\left[4500\mathrm{nl}/\mathrm{nM}/\mathrm{cm}\right]\left[1\mathrm{cm}\right] $$

Specific activity of β-galactosidase was calculated using the following formula:

$$ {\displaystyle \begin{array}{c}\mathrm{Specific}\kern0.17em \mathrm{activity}=\mathrm{nMole}\kern0.17em \mathrm{ONPG}\kern0.17em \mathrm{hydrolysed}/\mathrm{t}/\mathrm{mg}\;\mathrm{protein}.\\ {}\mathrm{t}=\mathrm{incubation}\kern0.17em \mathrm{time}\kern0.17em \mathrm{in}\kern0.17em \mathrm{minutes}\end{array}} $$

### Kinetics of mitochondrial membrane potential (ΔΨm/ MMP)

Time kinetics of DiOC_6_(3) uptake as indicator of alterations in membrane potential in NKE cells after treatment with mannan (20 μg/ml) was determined flowcytometrically (BD LSR II flow cytometer, BD Biosciences, USA). Autofluorescence from each cell suspension was acquired for 1 min and thereafter DiOC_6_(3) was added (5 nM final concentration) to determine dye uptake for 10 min at Ex λ 488 nm and Em λ 530 nm. The acquired data was analyzed using FlowJo V10.1 software and is representative of findings from three independent experiments.

To confirm the alterations in MMP in following various treatments NKE cells were stained with 100 nM JC-1 (final concentration) dye for 10 min (to correlate MMP changes with cyanine dye used for flowcytometry) and image were acquired using ZOE™ Fluorescent imager (Bio-Rad, USA) using both red and green channels LEDs. Images were merged using inbuilt software provided along with microscope.

#### Kinetics of endogenous ROS generation

The uptake and time dependent oxidation of CM-H_2_DCFDA in NKE cells after treatment with mannan (20 μg/ml; 15–240 min as indicated in results) was determined using flow cytometer and spectro-flurimetrically using SpectraMax M2 (Molecular devices microplate reader also. For flow cytometric acquisition, autofluorescence of cells was acquired for 1 min and thereafter CM-H_2_DCFDA (10 μM final conc.) was added to determine ROS dependent oxidation of dye till 10 min with continuous acquisition of fluorescence of oxidized dye using Ex λ 488 nm and Em λ 530 nm as described by Gupta, D. et al.*.* [27, 41]. The acquired data was analyzed using FlowJo V10.1 software and results are representative of findings from three independent experiments. Furthermore, fluorescence of oxidized dye was also acquired separately using microplate reader using Ex λ 488 nm and Em λ 530 nm following treatment with mannan as mentioned above. Results are representative of mean fluorescence and expressed as fold changes±SD of three independent experiments.

### Cytotoxicity, cell proliferation and cell survival studies

Exponentially growing (2.5 × 10^3^) cells were seeded and incubated for attachment (in 96 well plate) and thereafter treated with different concentrations of mannan (100 ng/ml to 5 mg/ml) to assess cytotoxicity in terms of proliferation. For radiation protection studies cells were seeded and treated with mannan (5 μg/ml to 40 μg/ml) for different time intervals (15 min, 30 min, 45 min and 60 min) followed by radiation exposure. Cells were incubated for 96 h post irradiation and thereafter processed for SRB assay as described by Vichai et al. with minor modifications [42]. Briefly, following various treatments and incubation, cells were washed with PBS to remove traces of FBS, and fixed by using 100 μl of 10% trichloroacetic acid (*w*/*v*); protein precipitation; incubation 1 h at 4°C). Thereafter plates were washed thrice with deionized water and air-dried. Samples were incubated for about 90 min at room temperature with SRB solution (50 μl/ well; 0.4% w/v in 1% acetic acid) and thereafter plates were washed thrice with 1% acetic acid to remove unbound SRB and air-dried at room temperature. The SRB bound with cellular proteins was extracted with 100 μ1 of 10 mM Tris base buffer (pH 10.5) and absorbance of SRB was measured by using spectrophotometer (BioTek, USA) at λ 565 nm and λ 690 nm as reference wavelength.

NKE cells were seeded (approx. 200 Cells/ plate) in 60 mm petri-plates and were allowed to attach for about 6–8 h in CO_2_ incubator for assessment of clonogenic efficacy as described by Franken et al.*.* with minor modification [43]. Briefly, plates were divided in different groups, treated as per groups made and incubated for 14 days to assess clonogenic efficacy. Fresh media was supplemented every forth day. After incubation colonies were fixed and stained with crystal violet [0.1% (w/v) in 70% (*v*/v) methanol]. Colonies with more than 50 cells were counted and plating efficiency (PE) and surviving fraction (SF) was determined as under:

PE = Colonies counted × 100/ cells plated.

SF = number of colonies counted/ [number of cells plated * (plating efficiency of control *100)].

### Radiation-mediated changes in surviving fraction of cells

NKE cells, NUB cells, HEK 293 cells, NKE ρ° cells, ACHN and A498 cells were seeded and were allowed to attach for about 6–8 h in CO_2_ incubator. Cells were irradiated with increasing dose of ionizing radiation (0–6 Gy) and clonogenic efficacy of respective cells was accessed as described by Franken et al. with minor modification [43]. After incubation visible colonies were counted and surviving fraction was calculated with respect to control. Results are expressed as fraction of cells surviving after treatment with respect to the control ±SD of three independent experiments.

### Generation of NKE ρ° cells from NKE cells

NKE ρ° cells were generated from NKE cells by selectively depleting mitochondrial DNA by prolonged exposure with ethidium bromide (50 ng/ml) as described by King et al [44]. Cells were maintained as indicated with additional supplementation of media with 100 μg/ml pyruvate and 50 μg/ml uridine [45]. Mitochondrial DNA was isolated from both NKE parental and ρ° cells using mitochondrial DNA isolation Kit (abcam, Cambridge, UK) according to manufacturer’s protocol. Cells were confirmed for absence of mitochondrial DNA (ND1 gene primers) using PCR and their auxotrophy for uridine were also confirmed.

#### Primer sequence

mt3187F:5‘-CTCAACTTAGTATTATACCC -3’.

mt4650R:5‘-GGAAATACTTGATGGCAGCT -3’.

The amplification was carried out using REDTaq ReadyMix according to manufacturer’s protocol (Sigma Aldrich, St Louis, MO). Briefly, 25 μl REDTaq ReadyMix, 5 μl of each primer (final concentration 10 μM), DNA template and 15 μl DEPC water were mixed gently. Reactions were amplified through 35 cycles at the following parameters: denaturation (94 °C for 1 min), annealing (52 °C for 2 min) and extension (72 °C for 3 min). Quantity and quality of the amplified DNA were measured by spectrophotometry at 280 nm and 230 nm using NanoDrop (Implen NanoPhotometer) and by agarose gel electrophoresis respectively.

### Reactive oxygen species (ROS) levels

The intracellular generation of reactive oxygen species (ROS) following different doses of radiation was measured in both NKE cells and NKE ρ° cells as described with minor modifications [41]. Briefly, cells were exposed to increasing dose of γ-radiation (sham irradiated, 2 Gy, 4 Gy and 6 Gy). Irradiated cells were incubated at 37°C in 5% CO_2_ and humid atmosphere for 24 h. Thereafter, cells were harvested and incubated with DHE (10 μM freshly prepared) in the dark for 15 min. The fluorescence from the oxidized dye was measured by flowcytometry at Ex λ 488 nm and Em λ 605 nm using BD LSR II flowcytometer (Becton, Dickinson, USA). The acquired data was analyzed using FlowJo V10.1 software and is representative of findings from three independent experiments. Results are also expressed as mean fluorescence ± SD (fold changes) and were calculated using graphpad prism 6.

### Mitochondrial membrane potential (ΔΨm; MMP)

The changes in ΔΨm of cells following various treatments was measured using DiOC_6_ (3) as previously described by Gupta et al [41]. Briefly, NKE cells and NKE ρ° cells were exposed to increasing dose of γ-radiation (sham, 2 Gy - 6 Gy) and followed by incubation till 24 h. Thereafter, cells were harvested and incubated with freshly prepared DiOC_6_ (3) solution (40 nM) for 15 min in dark. The fluorescence of DiOC_6_ (3) was acquired at Ex λ 488 nm and Em λ 530 nm using BD LSR II flowcytometer (Becton, Dickinson, USA). The acquired data was analyzed using FlowJo V10.1 software and is representative of findings from three independent experiments. Results are also represented as mean fluorescence ± SD (fold changes) were calculated using graph pad prism 6.

### Alterations in cardiolipin content, exclusive inner mitochondrial membrane phospholipid

Alterations of mitochondrial cardiolipin content following various treatments to cells was evaluated as NAO dye incorporation. After 24 h of various treatments exposure with γ-radiation and/or treated with mannan (20 μg/ml), 30 min prior to radiation (3 Gy), cells were trypsinized and washed with PBS. After washing cell pellets were suspended in RPMI 1640 (without phenol red and FBS) and stained with NAO (100 nM; final conc.), for 30 min at 37 °C in dark and fluorescence were acquired flowcytometrically at Ex λ 488 nm and Em λ 530 nm [26]. The data were analyzed using FlowJo V10.1. The data presented is representative of findings from three independent experiments.

### Western blotting

Expression of SOD I (Cu/ZnSOD) and SOD II (MnSOD) was measured by immune-blotting technique 24 h post treatment as described by Gupta et al with minor modifications. To assess the role of stress responsive pathways, the phosphorylation of JNK and p38 was also measured following various treatments at different time intervals [27, 41, 46]. Briefly, logarithmically growing NKE parental as well as rho zero cells were treated with mannan and/ or exposed to γ-radiation. Cells were washed with ice-cold PBS, and thereafter lysed in RIPA buffer containing protease and phosphatase inhibitors [PMSF, sodium orthovanadate, protease and phosphatase inhibitor cocktail (Sigma Aldrich, USA), sodium fluoride]. Samples were vortexed at 4 °C for 20 min followed by centrifugation (12,000 g, 4 °C for 20 min) and collection of supernatant for western blotting. Protein estimation was performed using BCA reagent (Sigma Aldrich, USA) and samples were loaded in equal quantity (40 μg protein/ well) for separation on 8–20% gradient SDS-polyacrylamide gel. After gel electrophoresis proteins were transferred into PVDF membrane (Amersham, GE healthcare, Germany), using Tris-glycine transfer buffer containing 10% Methanol. After transfer, membranes were blocked using 5% skimmed milk (in TBST) for 1 h and thereafter the membranes were incubated overnight (at 4 °C) with anti SOD I (1:1000), anti SOD II (1:1000), anti phospho JNK (1:1000), anti phosho p38 (1:1000) and anti ß-actin (1:5000) antibody in 2% skimmed milk (TBST). The membranes were washed thrice using TBST to remove unbound primary antibody and incubated with appropriate secondary antibodies conjugated with horseradish peroxidase (HRP) for 3 h. The expression of proteins was measured using super signal West Pico chemiluminescent substrate (Thermo Scientific, USA).

### Data analyses and statistical evaluations

All experiments were performed at least three times independently. For the graphical representation of the data, y-axis error bars represents mean ± SD. Statistical analysis of data was done using one-way analysis of variance (ANOVA) followed post hoc analysis using Tukey’s multiple comparisons test (Prism 6.0, GraphPad Software, San Diego, CA, USA) unless otherwise stated. Differences were designated significant at values * *p* < 0.05 and were labeled with asterisks.

## Abbreviations

ARS: Acute radiation syndrome
DHE: Dihydroethidine
ETC: Electron transport chain
JC-1: 5,5′,6,6′-tetrachloro-1,1′,3,3′-tetraethylbenzimidazolylcarbocyanine iodide
MMP or ΔΨm: Mitochondrial membrane potential
MOS: Mannan oligosaccharide
NAO: 3, 3’-DihexyloxacarbocyanineIodide [DiOC_6_ (3)], 5-(and-6)-chlormethyl 2′,7’dichlorodihydrofluorescein diacetate acetyl ester [CM-H_2_DCFDA], 10-N-nony1–3, 6-bis [dimethylamino] acridine
NKE: Human normal kidney epithelial
NUB: cells human normal urinary bladder cells
ROS: Reactive oxygen species
SRB: Sulphorhodamine-B
TLR: Toll like receptor
